# Hyperactivated Wnt-β-catenin signaling in the absence of sFRP1 and sFRP5 disrupts trophoblast differentiation through repression of Ascl2

**DOI:** 10.1186/s12915-020-00883-4

**Published:** 2020-10-27

**Authors:** Haili Bao, Dong Liu, Yingchun Xu, Yang Sun, Change Mu, Yongqin Yu, Chunping Wang, Qian Han, Sanmei Liu, Han Cai, Fan Liu, Shuangbo Kong, Wenbo Deng, Bin Cao, Haibin Wang, Qiang Wang, Jinhua Lu

**Affiliations:** 1 Reproductive Medical Center, The First Affiliated Hospital of Xiamen University, Xiamen, 361003 Fujian People’s Republic of China; 2 Fujian Provincial Key Laboratory of Reproductive Health Research, School of Medicine, Xiamen University, Xiamen, 361102 Fujian People’s Republic of China; 3 State Key Laboratory of Stem Cell and Reproductive Biology, Institute of Zoology, Chinese Academy of Sciences, Beijing, 100101 People’s Republic of China; 4 Department of Surgery, The Ohio State University Wexner Medical Center, Ohio 43210 Columbus, USA

**Keywords:** *Sfrp1* and *Sfrp5*, Hyperactivation, Canonical Wnt pathway, Trophoblast, *Ascl2*

## Abstract

**Background:**

Wnt signaling is a critical determinant for the maintenance and differentiation of stem/progenitor cells, including trophoblast stem cells during placental development. Hyperactivation of Wnt signaling has been shown to be associated with human trophoblast diseases. However, little is known about the impact and underlying mechanisms of excessive Wnt signaling during placental trophoblast development.

**Results:**

In the present work, we observed that two inhibitors of Wnt signaling, secreted frizzled-related proteins 1 and 5 (Sfrp1 and Sfrp5), are highly expressed in the extraembryonic trophoblast suggesting possible roles in early placental development. Sfrp1 and Sfrp5 double knockout mice exhibited disturbed trophoblast differentiation in the placental ectoplacental cone (EPC), which contains the precursors of trophoblast giant cells (TGCs) and spongiotrophoblast cells. In addition, we employed mouse models expressing a truncated β-catenin with exon 3 deletion globally and trophoblast-specifically, as well as trophoblast stem cell lines, and unraveled that hyperactivation of canonical Wnt pathway exhausted the trophoblast precursor cells in the EPC, resulting in the overabundance of giant cells at the expense of spongiotrophoblast cells. Further examination uncovered that hyperactivation of canonical Wnt pathway disturbed trophoblast differentiation in the EPC via repressing Ascl2 expression.

**Conclusions:**

Our investigations provide new insights that the homeostasis of canonical Wnt-β-catenin signaling is essential for EPC trophoblast differentiation during placental development, which is of high clinical relevance, since aberrant Wnt signaling is often associated with trophoblast-related diseases.

## Background

The placenta, forming the fetal-maternal interface, is essential for the survival and growth of the fetus in eutherian mammals [1, 2]. In mice, a mature placenta consists of three trophoblast layers: the outermost giant cell layer, the intermediate spongiotrophoblast layer, and the innermost labyrinth layer. The trophoblast cells of the placenta arise from the outer trophectoderm of the blastocyst. After implantation (E4.5), while the mural trophectoderm cells stop dividing but keep endoreduplication to form the primary trophoblast giant cells invading into the uterus, the polar trophectoderm cells maintain proliferating and form diploid extraembryonic ectoderm (ExE) and ectoplacental cone (EPC), from the outer regions of which more TGCs form. Subsequently, the ExE form the chorion layer which gives rise to the labyrinth including two syncytiotrophoblast layers, while the EPC develops into the spongiotrophoblast layer [1]. The balanced differentiation of various trophoblast cell types is a prerequisite for normal placentation [3, 4]. The achaete-scute complex homolog-like 2 (*Ascl2*, also *Mash2*), one member of the basic helix–loop–helix (bHLH) family, has been shown to play a critical role during EPC development. *Ascl2* is located in the EPC and diminishes as trophoblast cells differentiate into TGCs. Ablation of *Ascl2* led to embryonic lethality owing to defective spongiotrophoblast formation [5]. Moreover, *ASCL2* is reported to be expressed in human freshly isolated cytotrophoblasts [6] and proximal column extravillous trophoblasts [7], corresponding to the EPC and/or spongiotrophoblast layer in mice. However, the signals that affect EPC trophoblast differentiation and how they interact with *Ascl2* remain largely unknown.

Previous studies have provided evidence that Wnt signaling is a critical player during placental development [8–15]. *Wnt7b* null mice died at mid-gestation stage due to defective chorioallantoic fusion [10]. Targeted disruption of *Wnt2* caused impaired labyrinth [11]. *Fzd5* mutation was reported to disturb placental labyrinth development [12], and our previous work further demonstrated the necessity of canonical Wnt2-Fzd5-Gcm1 (Glial cells missing-1) signaling for chorioallantoic branching and trophoblast syncytialization during placentation [9]. Recently, Wnt signal was shown to be essential for the derivation and maintenance of human trophoblast stem cells (HTSCs) and organoids [16–18]. Although Wnt signaling is essential for normal placental development, hyperactivation of Wnt signaling are often observed in human trophoblast-related diseases such as complete hydatidiform moles and choriocarcinoma [8, 19]. However, whether and how excessive Wnt signaling affects EPC trophoblast differentiation remains largely unclear.

In the present work, we employed a variety of genetic mouse models and cultured trophoblast cells to demonstrate that hyperactivation of canonical Wnt pathway leads to overabundance of TGCs at the expense of spongiotrophoblast cells, via repressing *Ascl2* during EPC trophoblast differentiation, providing direct genetic evidence for the crucial role of suitable canonical Wnt signaling during placental trophoblast differentiation.

## Results

### *Sfrp1* and *Sfrp*5 deficiency leads to excessive TGC differentiation and compromised placental development

Secreted frizzled-related proteins (SFRPs), containing a cysteine-rich domain (CRD) that is 30 to 50% similar in sequence to that of the frizzled protein while lacking the transmembrane domain, serve mainly as extracellular inhibitors of Wnt signaling by directly blocking the interactions between the Wnt and Frizzled receptors [20]. Sequence comparison and phylogenetic analysis reveal that SFRP1, SFRP2, and SFRP5 are closely related [21]. To define the potential functions of these SFRPs during placentation, we firstly examine the expression profile of *Sfrp1*, *Sfrp2*, and *Sfrp5* during early placental development. Through whole-mount in situ hybridization, we observed that both *Sfrp1* and *Sfrp5* were expressed in the extraembryonic tissues at E7.5 and E8.5, except *Sfrp2* (Additional file 1: Fig. S1A). Further examination of the placental sections at E7.5-E9.5 showed that the expression of *Sfrp1* and *Sfrp5* was abundant in extraembryonic trophoblast, including trophoblast cells in the chorion and EPC, as well as the subsequent spongiotrophoblast layer (Additional file 1: Fig. S1B). These findings suggest the potential roles of *Sfrp1* and *Sfrp5* during early placental development.

**Fig. 1:**
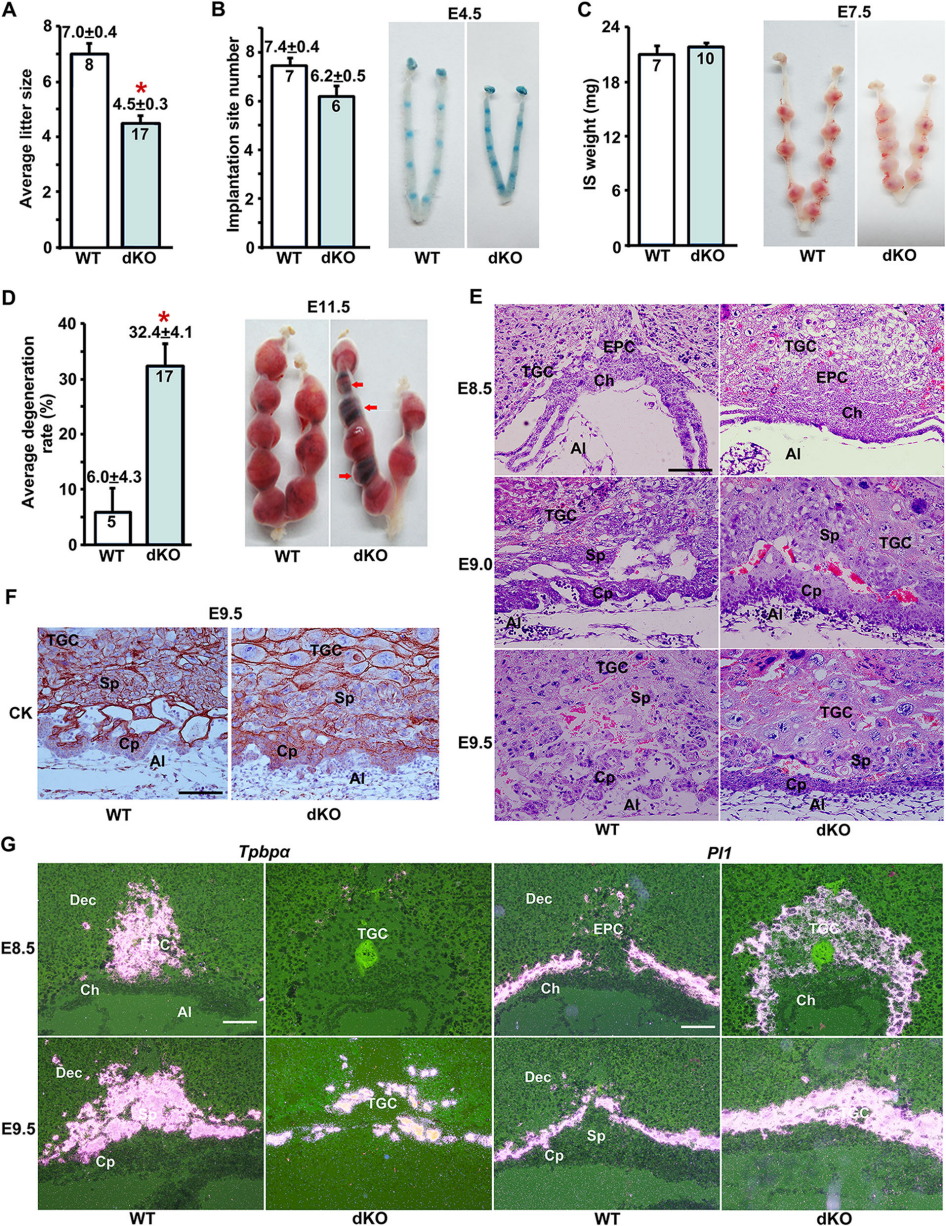
*Sfrp1* and *Sfrp5* deficiency leads to excessive TGC differentiation and compromised placental development. **a** Average litter sizes of WT as well as *Sfrp1* and *Sfrp5* double knockout (dKO) mice. *P < 0.05. **b** Average number of implantation sites and implantation status of WT and dKO mice at E4.5. **c** Average weight of implantation sites and implantation status of WT and dKO mice at E7.5. **d** Average embryo degeneration rate and representative uteri of WT and dKO mice at E11.5. Arrows indicated degenerated embryos. *P < 0.05. **e** Hematoxylin and eosin (HE) staining of E8.5–E9.5 placental sections of WT and dKO mice. **f** Immunostaining analysis of cytokeratin (CK) on E9.5 placental sections of WT and dKO mice. **g** In situ hybridization analysis of the expression of *Tpbpα* and *Pl1* in the placentas of WT and dKO mice, at E8.5 and E9.5. In **a**–**d**, numbers within the bars indicated the number of pregnant mice examined. Images in **e**–**g** are representatives of at least three independent experiments. Al, allantois; Ch, chorion; Cp, chorionic plate; Sp, spongiotrophoblast; TGC, trophoblast giant cell. Scale bar, 100 μm

To assess the physiological relevance of *Sfrp1* and *Sfrp5* during placental development, we examined the pregnancy outcome of *Sfrp1* and *Sfrp5* double knockout (dKO) females crossed with dKO males, and observed that the average litter size was significantly reduced, compared with that of the wildtype (WT) intercrosses (4.5 ± 0.3 vs 7.0 ± 0.4, *P < 0.05, Fig. 1a). We subsequently analyzed the stage-specific effect of *Sfrp1* and *Sfrp5* during pregnancy. As illustrated in Fig. 1b, normal embryo implantation exhibited by blue bands was observed in both dKO and WT mice at E4.5, and the average number of implantation sites in dKO mice was comparable to that of the WT mice (6.2 ± 0.5 vs 7.4 ± 0.4). Moreover, *Sfrp1* and *Sfrp5* deficiency did not hamper uterine decidualization, displayed by the normal weight and size of implantation sites at E7.5 (21.84 ± 0.47 mg vs 21.01 ± 0.94 mg, Fig. 1c). However, increased rate of embryo degeneration was observed in dKO mice at E11.5, (32.4 ± 4.1% vs 6.0 ± 4.3%, *P < 0.05, Fig. 1d). Further histological examination and immunostaining analysis of cytokeratin (CK) marking placental trophoblast cells revealed impaired differentiation of extraembryonic trophoblast lineage with expanded TGCs and reduced spongiotrophoblast layer as well as compact chorion, at E8.5–E9.5 (Fig. 1e, f, and Additional file 1: Fig. S2A). In addition, we performed in situ hybridization to examine the expression of marker genes placental lactogen I (*Pl1*), specifically expressed in TGCs, and trophoblast-specific protein α (*Tpbpα)*, marking EPC and/or spongiotrophoblast cells. While *Tpbpα*^+^ trophoblast cells were decreased, *Pl1-*expressing trophoblast cells were significantly increased in dKO mice (Fig. 1g). These data reveal that *Sfrp1* and *Sfrp5* deletion disturbs EPC trophoblast differentiation with excessive TGCs during early placentation.

### *Sfrp1* and *Sfrp*5 deletion renders hyperactivation of canonical Wnt signaling and disturbs trophoblast differentiation in the EPC

**Fig. 2:**
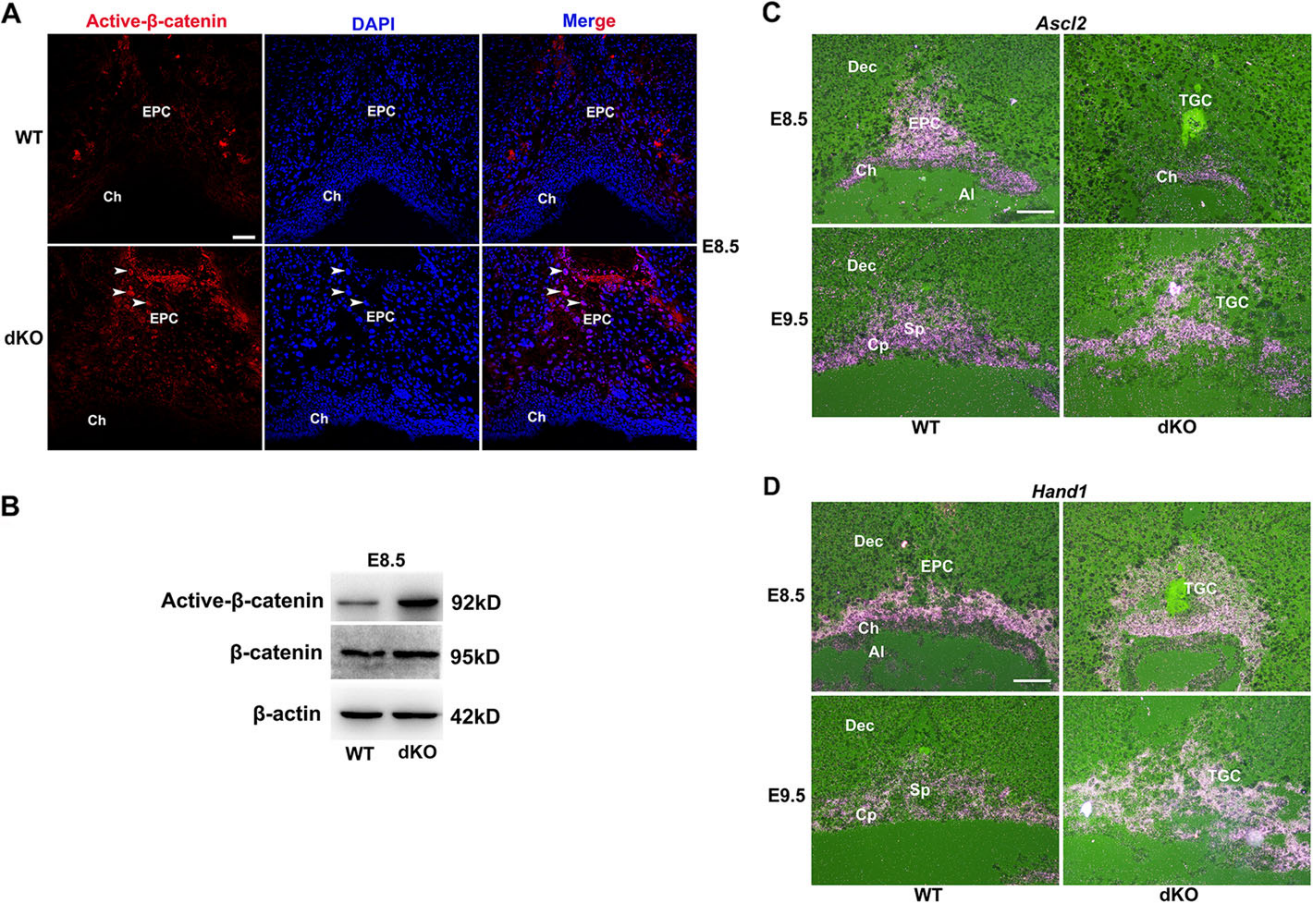
*Sfrp1* and *Sfrp5* deletion renders hyperactivation of canonical Wnt signaling and disturbs EPC trophoblast differentiation. **a**, **b** Immunostaining and western blot analysis of active-β-catenin in WT and dKO placentas at E8.5. Cy3-labeled β-catenin in red, DAPI-labeled nuclei in blue. Arrowheads indicated trophoblast cells with nuclear localization of active-β-catenin. β-actin served as the internal control. **c**, **d** The expression of *Ascl2* and *Hand1* was revealed by in situ hybridization in the WT and dKO placentas at E8.5 and E9.5. Images in **a**, **c**, and **d** are representatives of at least three independent experiments. Al, allantois; Dec, decidua; EPC, ectoplacental cone; Ch, chorion; Cp, chorionic plate; Sp, spongiotrophoblast; TGC, trophoblast giant cell. Scale bar, 100 μm

Since *Sfrp1* and *Sfrp5* regulate both the canonical and noncanonical Wnt pathway, we firstly detected the activity of canonical Wnt pathway. We observed that the nuclear localization of active-β-catenin, an indicator of canonical Wnt signaling activity, was increased significantly in the EPC and TGCs of dKO placentas at E8.5 (Fig. 2a and Additional file 1: Fig. S2B), which was further confirmed by western blot analysis (Fig. 2b). These results suggest that the absence of *Sfrp1* and *Sfrp5* enhanced the activity of canonical Wnt signaling. Considering the phenotype of reduced EPC trophoblast cells and increased number of TGC in dKO placentas, we examined the expression of the key genes essential for trophoblast cell differentiation in the EPC. The genes *Ascl2* and *Hand1* (heart and neural crest derivatives expressed transcript 1), encode two transcription factors of the basic helix-loop-helix (bHLH) family. *Ascl2* is required to maintain the EPC progenitor population, determines the specification of *Tpbpα*-positive trophoblast cells, and inhibits *Pl1*-expressing giant cell differentiation, while Hand1 has opposing roles and promotes *Pl1*-expressing TGC differentiation [22]. Ablation of *Ascl2* or *Hand1* in mice both lead to embryo lethality owing to defective placental development [5, 23, 24]. By in situ hybridization, we found that while *Ascl2*-positive trophoblast cells were decreased (Fig. 2c), the number of *Hand1*-expression TGCs were increased significantly (Fig. 2d), in dKO mice at E8.5 and E9.5, similar to that of *Ascl2* mutant mice [5]. These findings illustrate that loss of *Sfrp1* and *Sfrp5* increases the canonical Wnt signaling activity which might disturb the differentiation of trophoblast progenitors in the EPC.

### Global stabilization of β-catenin leads to impaired placental development with excessive TGC differentiation

**Fig. 3:**
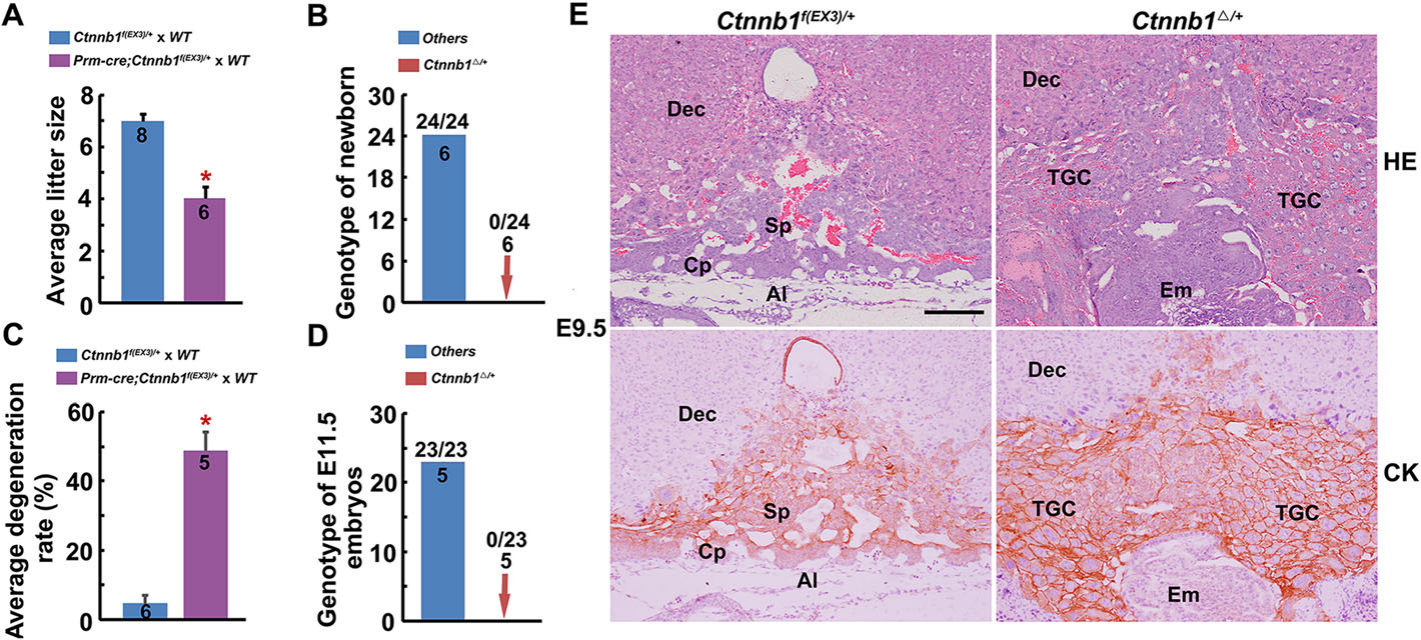
Global stabilization of β-catenin leads to impaired placental development with excessive TGC differentiation. **a** Average litter sizes of WT females mated with *Ctnnb1*^*f(EX3)/+*^ and *Prm-cre*; *Ctnnb1*^*f(EX3)/+*^ males, respectively. *P < 0.05. **b** Genotyping of the newborns from WT females mated with *Prm-cre*; *Ctnnb1*^*f(EX3)/+*^ males. **c** Average embryo degeneration rate of WT females mated with *Ctnnb1f*^*(Ex3)/+*^ and *Prm-cre*; *Ctnnb1*^*f(EX3)/+*^ males, respectively, at E11.5. *P < 0.05. **d** Genotyping of the remaining survived embryos from WT female mated with *Prm-cre*; *Ctnnb1*^*f(EX3)/+*^ male mice at E11.5. **e** HE and CK staining of *Ctnnb1*^*f(EX3)/+*^ and *Ctnnb1*^*fΔ/+*^ placental sections at E9.5. In **a**–**d**, numbers within and above the bars indicated the number of pregnant mice and embryos examined, respectively. Images in **e** are representatives of at least three independent experiments. Al, allantois; Dec, decidua; Cp, chorionic plate; Em, embryo; Sp, spongiotrophoblast; TGC, trophoblast giant cell. Scale bar, 100 μm

Since *Sfrp1* and *Sfrp5* deletion led to exaggerated canonical Wnt signaling activity, to further define whether the hyperactivation of canonical Wnt signaling disturbed EPC trophoblast differentiation, we introduced the *Ctnnb1*^*f(EX3)/f(EX3)*^ mouse model, in which the exon 3 of β-catenin gene is flanked by loxp sites, and Cre recombinase-mediated excision would give rise to the expression of a stabilized, constitutively active form of β-catenin resistant to degradation by the GSK3β-mediated proteasome pathway [25]. Through crossing the *Ctnnb1*^*f(EX3)/f(EX3)*^ male with *Prm-cre* (functioning in adult testis) transgenic female, *Prm-cre*; *Ctnnb1*^*f(EX3)/+*^ male mice were generated. When crossing WT females with *Prm-cre*; *Ctnnb1*^*f(EX3)/+*^ males, the *Ctnnb1*^*Δ/+*^ conceptus with stabilized β-catenin and hyperactivated canonical Wnt pathway were generated. In this breeding, the average litter size decreased significantly, and *Ctnnb1*^*Δ/+*^ pups failed to be born (Fig. 3a, b) and degenerated before E11.5 (Fig. 3c, d). Moreover, histological examination and immunostaining analysis of CK showed excessive TGCs in the *Ctnnb1*^*Δ/+*^ conceptus at E9.5 (Fig. 3e), with increased activity of Wnt signaling (Additional file 1: Fig. S3), similar to the phenotype of *Sfrp1, 5* dKO mice. Further examination indicated that the defects of the *Ctnnb1*^*Δ/+*^ conceptus appeared as early as E7.5 (Additional file 1: Fig. S4). These data demonstrate that stabilized β-catenin disturbs placental development with excessive TGC differentiation.

### Trophoblast-specific stabilization of β-catenin impairs EPC trophoblast differentiation with excessive TGC differentiation

**Fig. 4:**
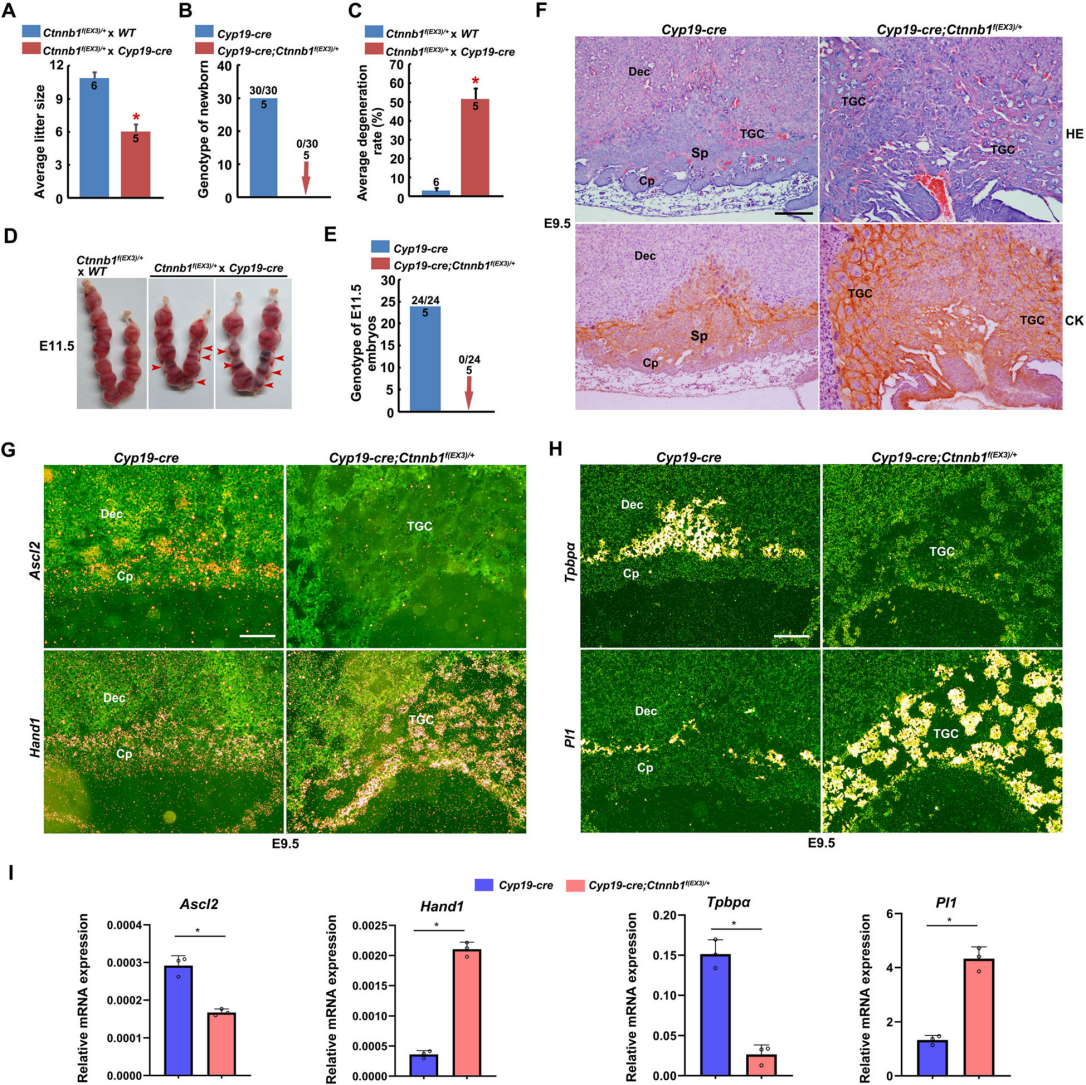
Trophoblast-specific stabilization of β-catenin impairs EPC trophoblast differentiation with excessive TGC differentiation. **a** Average litter sizes of WT and *Cyp19-cre* female mated with *Ctnnb1*^*f(EX3)/+*^ male mice, respectively. *P < 0.05. **b** Genotyping of the newborns from the intercrosses of *Cyp19-cre* female and *Ctnnb1*^*f(EX3)/+*^ male mice. **c**, **d** Average embryo degeneration rate and representative uteri of WT and *Cyp19-cre* female mated with *Ctnnb1*^*f(EX3)/+*^ male mice, respectively, at E11.5. Arrowheads indicated degenerated embryos. *P < 0.05. **e** Genotyping of the remaining survived embryos from the intercrosses of *Cyp19-cre* female and *Ctnnb1*^*f(EX3)/+*^ male mice at E11.5. **f** HE and CK staining of *Cyp19-cre* and *Cyp19-cre*; *Ctnnb1*^*f(EX3)/+*^ placental sections at E9.5. **g**–**i** The expression of *Ascl2*, *Hand1*, *Tpbpα* and *Pl1* in the *Cyp19-cre* and *Cyp19-cre*; *Ctnnb1*^*f(EX3)/+*^ placentas at E9.5, were detected by in situ hybridization and qRT-PCR, respectively. Values are normalized by *Gapdh* expression level and indicated as means ± SD. *N* = 3. *P < 0.05. In **a**–**c** and **d**, numbers within and above the bars indicated the number of pregnant mice and embryos examined, respectively. Images in **f**–**h** are representatives of at least three independent experiments. Dec, decidua; Cp, chorionic plate; Sp, spongiotrophoblast; TGC, trophoblast giant cell. Scale bar, 100 μm

To further verify the contributions of trophoblast-specifically stabilized β-catenin protein, we generated the *CYP19*-*cre*; *Ctnnb1*^*f(EX3)/+*^ mouse model, in which β-catenin protein is dominant-stabilized specifically in extraembryonic trophoblast cells. When *CYP19*-*cre* females were crossed with *Ctnnb1*^*f(EX3)/+*^ males, the average litter size was decreased significantly, and the *CYP19*-*cre*; *Ctnnb1*^*f(EX3)/+*^ offspring failed to be born (Fig. 4a, b) and degenerated before E11.5 (Fig. 4c–e). Histological analysis and immunostaining analysis of CK exhibited abnormal extraembryonic tissues with excessive TGCs differentiation in *CYP19*-*cre*; *Ctnnb1*^*f(EX3)/+*^ conceptus (Fig. 4f), which might be responsible for the embryo degeneration at mid-gestation and recapitulated the phenotypes of the *Sfrp1, 5* dKO and *Ctnnb1*^*Δ/+*^ mice. Moreover, while the number of *Ascl2*- and *Tpbpα*- positive trophoblast cells decreased, *Hand1*- and *Pl1*-expressing TGCs were increased significantly in the *CYP19*-*cre*; *Ctnnb1*^*f(EX3)/+*^ placentas at E9.5 (Fig. 4g, h). Quantitative RT-PCR (qRT-PCR) analysis also showed decreased expression of *Ascl2* and *Tpbpα* and increased expression of *Hand1* and *Pl1* in *CYP19*-*cre*; *Ctnnb1*^*f(EX3)/+*^ placentas (Fig. 4i). These findings suggest that trophoblast cells in the EPC might differentiate excessively into TGCs in the presence of stabilized β-catenin. In addition, we observed increased intensity of nucleus-localized active-β-catenin in trophoblast cells of the EPC and TGCs of *CYP19*-*cre*; *Ctnnb1*^*f(EX3)/+*^ conceptus (Additional file 1: Fig. S5), consistent with the findings in the *Sfrp1* and *Sfrp5* mutant mice. In summary, trophoblast-specific stabilization of β-catenin induced hyperactivation of canonical Wnt pathway which impairs EPC trophoblast differentiation.

### Hyperactivation of canonical Wnt signaling disturbs EPC trophoblast differentiation via repressing *Ascl2* expression

**Fig. 5:**
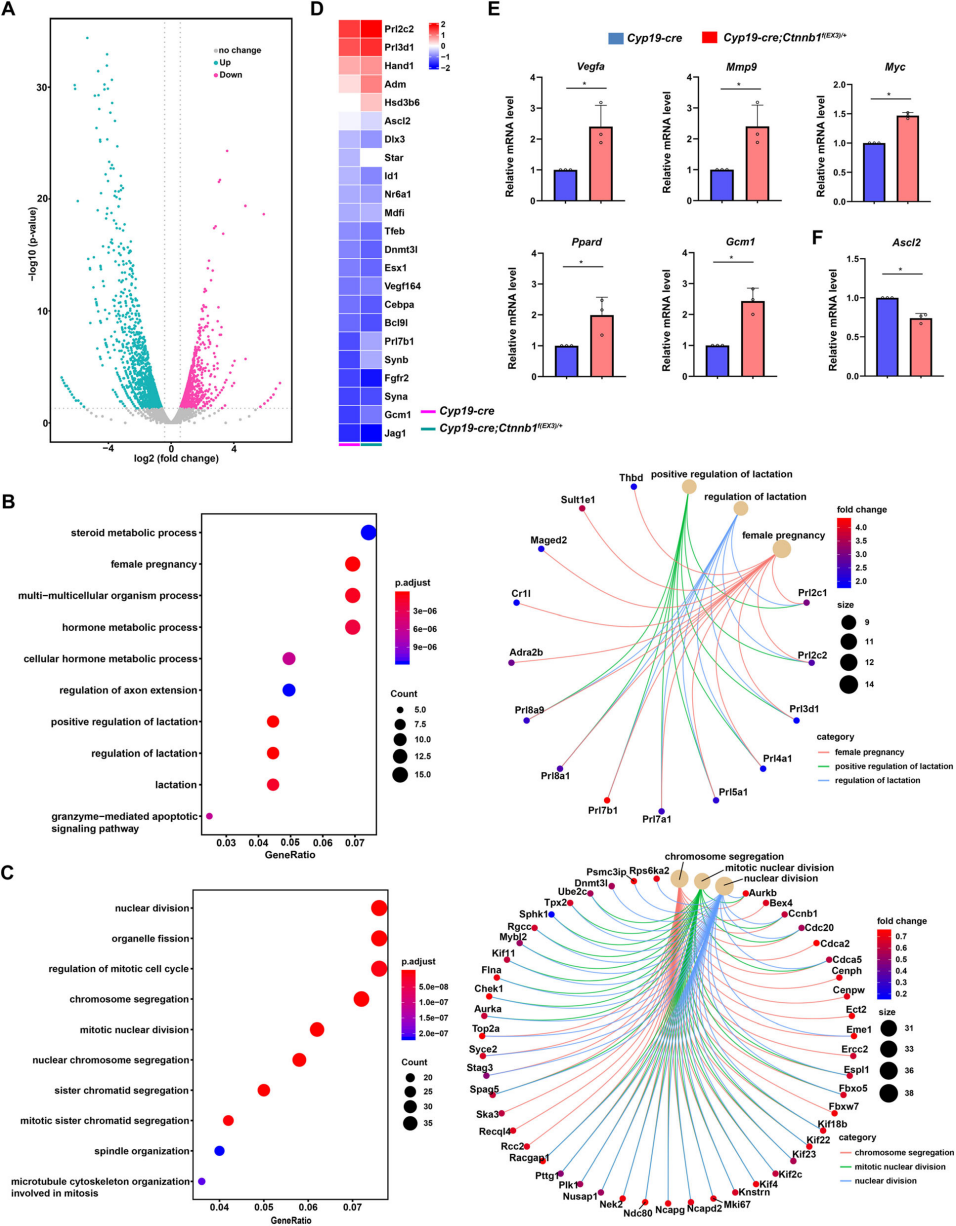
Hyperactivation of canonical Wnt signaling disturbs EPC trophoblast differentiation via repressing *Ascl2* expression. **a** Volcano plot displaying differentially expressed genes (DEG) between the *Cyp19-cre* and *Cyp19-cre*; *Ctnnb1*^*f(EX3)/+*^ placentas (fold change > 1.5, *P* value < 0.05). **b**, **c** GO enrichment analysis of increased genes (**b**) and decreased genes (**c**) between *Cyp 19-cre* and *Cyp19-cre*; *Ctnnb1*^*f(EX3)/+*^ placentas. **d** Heatmap showing the expression of trophoblast-located genes in the *Cyp19-cre* and *Cyp19-cre*; *Ctnnb1*^*f(EX3)/+*^ placentas. **e**, **f** The expression of Wnt target genes and *Ascl2* was confirmed by qRT-PCR. Values are normalized by *Gapdh* expression level and indicated as means ± SD. *N* = 3. *P < 0.05

To dissect the underlying mechanism by which hyperactivated canonical Wnt signaling influences trophoblast differentiation, we preformed RNA-seq on *CYP19*-*cre* and *CYP19*-*cre*; *Ctnnb1*^*f(EX3)/+*^ placentas at E8.5. 209 upregulated genes and 548 downregulated genes were observed in the *CYP19*-*cre*; *Ctnnb1*^*f(EX3)/+*^ placentas (fold change > 1.5, *P* value < 0.05, Fig. 5a, Additional file 2: Table S2). Gene ontology (GO) enrichment analysis revealed that the upregulated genes, including *Prl2c2*, *Prl3d1*, and *Prl7b1*, were related to the female reproduction and the regulation of lactation (Fig. 5b), possibly due to the increased number of TGCs expressing and/or secreting these molecules; the downregulated genes were related to cell cycle and cell division (Fig. 5c and Additional file 1: Fig. S6), which might be resulted from the excessive differentiation of TGCs without nuclear and cytoplasmic division. Moreover, the expression of genes located in different trophoblast cell types were examined in the *CYP19*-*cre* and *CYP19*-*cre*; *Ctnnb1*^*f(EX3)/+*^ placentas (Additional file 2: Table S3), and their expression patterns were consistent with the placental defects (Fig. 5d). In addition, we observed that several Wnt target genes, such as *Vegfa*, *Mmp9*, *Myc*, *Ppard*, and *Gcm1*, were upregulated in *CYP19*-*cre*; *Ctnnb1*^*f(EX3)/+*^ placentas (Additional file 2: Table S3), which was further confirmed by qRT-PCR analysis (Fig. 5e), indicating the enhanced activity of canonical Wnt signaling.

As a marker of progenitor trophoblast cells in the EPC, *Ascl2* also functions as an important transcription factor essential for the maintenance of the EPC, since *Ascl2* deletion results in increased number of giant cells at the expense of the EPC layer [5], similar to the phenotypes in our present study. Moreover, *Ascl2* was reported to be a target of canonical Wnt signaling in intestinal neoplasia and intestinal stem cells [26, 27]. Indeed, according to our RNA-Seq data, A*scl2* expression was decreased in the placenta with hyperactivated canonical Wnt signaling, which was further confirmed by qRT-PCR analysis (Fig. 5f). Based on the above observations, we speculated that *Ascl2* might be a target of canonical Wnt signaling during EPC trophoblast differentiation.

### Excessive canonical Wnt signaling activity restrains spongiotrophoblast differentiation through suppressing *Ascl2* in cultured trophoblast cells

**Fig. 6:**
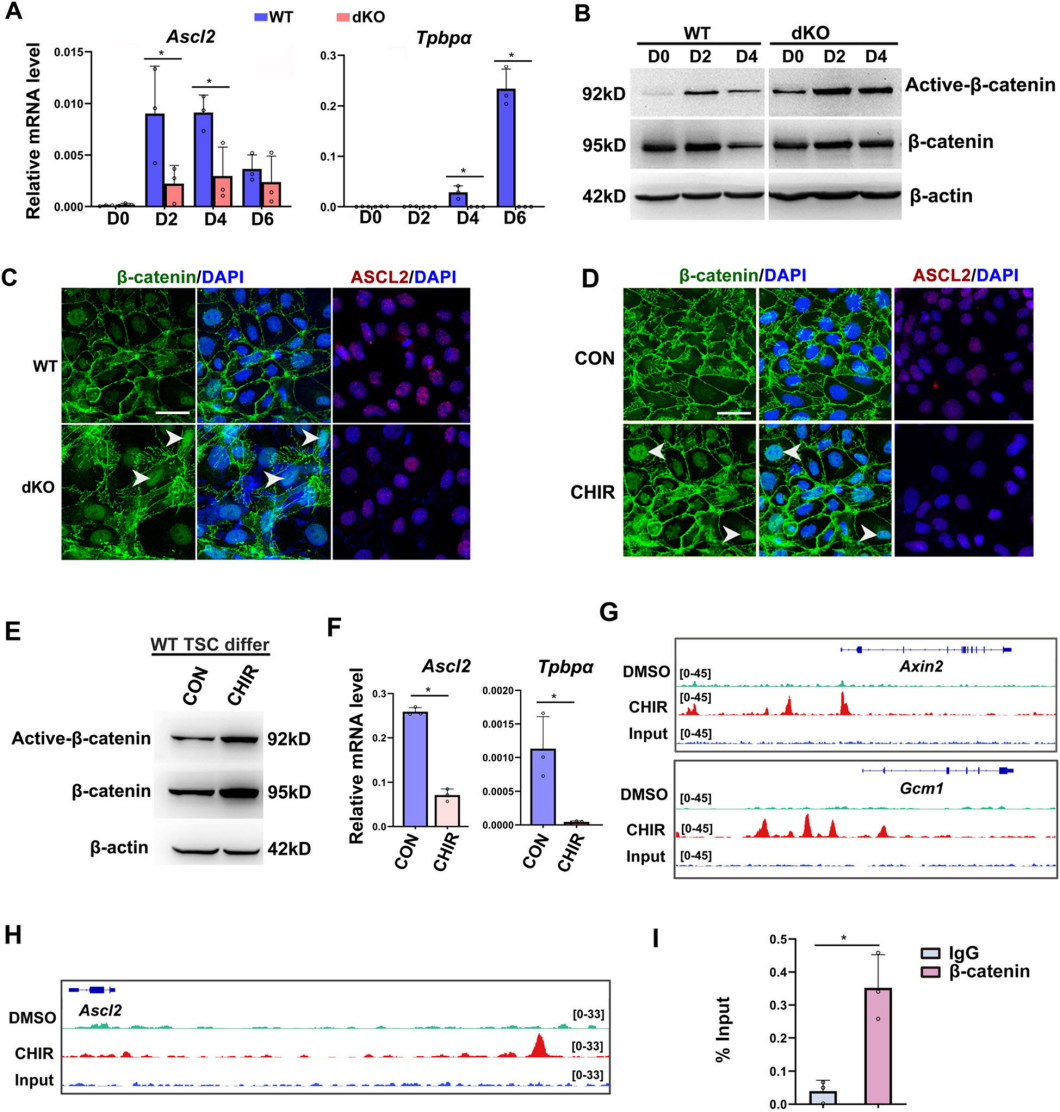
Excessive canonical Wnt signaling activity restrains spongiotrophoblast differentiation through suppressing *Ascl2* in cultured trophoblast cells. **a** The expression of *Ascl2* and *Tpbpα* was analyzed by qRT-PCR in WT as well as *Sfrp1* and *Sfrp5* dKO trophoblast differentiated for D0-D6. *N* = 3. *P < 0.05. **b** Western blot analysis of active-β-catenin during WT and dKO trophoblast differentiation. **c** Immunostaining of β-catenin (green) and ASCL2 (red) in WT and dKO trophoblast cells at D4. Arrowheads indicated nucleus-located β-catenin. DAPI-labeled nuclei in blue. **d**, **e** Immunostaining of β-catenin (green) and ASCL2 (red), and western blot analysis of active-β-catenin in the presence or absence of CHIR at D4. Arrowheads indicated nucleus-located β-catenin. DAPI-labeled nuclei in blue. **f** The expression of *Ascl2* and *Tpbp*α was detected by qRT-PCR in the presence of CHIR or not, at D4. *N* = 3. *P < 0.05. **g**–**i** After treatment with CHIR for 36 h, ChIP-seq showing β-catenin enrichment at Wnt targeted gene loci (Axin2 and Gcm1) (**g**), and the locus about 20 kb upstream of Ascl2 promoter (**h**), which is confirmed by ChIP-qPCR analysis (**i**). *N* = 3. *P < 0.05. β-actin served as the internal control in **b**, **e**. Values are normalized by *Gapdh* expression level and indicated as means ± SD in **a**, **f**, **i**. Images in **c**, **d** are representatives of at least three independent experiments. Scale bar, 100 μm

In order to verify the hypothesis that hyperactivation of canonical Wnt signaling disturbs the EPC differentiation via repressing *Ascl2* expression, we further employed in vitro cultured trophoblast stem (TS) cell lines, which undergo differentiation in the absence of FGF4 and mitomycin C-treated mouse embryonic fibroblast-conditioned medium (FCM) [28]. Compared with WT TS cells, *Sfrp1* and *Sfrp5* dKO TS cells showed decreased expression of *Ascl2* and *Tpbpα* during differentiation (Fig. 6a, c), consistent with the in vivo observations. Moreover, the level of active-β-catenin protein (Fig. 6b) and the nuclear localization of active-β-catenin (Fig. 6c) were increased significantly in the absence of *Sfrp1* and *Sfrp5*. TOP-Flash assay further confirmed hyperactivated β-catenin transcription activity in *Sfrp1* and *Sfrp5* dKO TS cells (Additional file 1: Fig. S7A). To confirm the contributions of hyperactivated canonical Wnt pathway to disturbed trophoblast cell differentiation, we employed CHIR99021 (CHIR), the agonist of canonical Wnt pathway [29]. After treatment with CHIR, the nuclear localization of β-catenin was enhanced (Fig. 6d), and the levels of active-β-catenin protein were increased remarkably (Fig. 6e). Meanwhile, the expression level of *Ascl2* and *Tpbpα* were decreased significantly (Fig. 6d, f). On the contrary, *Sfrp1* and *Sfrp5* dKO TS cells treated with XAV939, an inhibitor of Wnt pathway, or with *Ascl2* overexpression exhibited increased expression of *Tpbpα* and decreased *Pl1* expression (Additional file 1: Fig. S7B and C). These findings demonstrate that excessive canonical Wnt signaling represses *Ascl2* expression which is responsible for the disturbed trophoblast differentiation.

Generally, upon the activation of canonical Wnt pathway, stabilized β-catenin translocate into the nucleus, where it serves as a coactivator of the TCF/LEF DNA binding factors, to activate the transcription of target genes. In colorectal cancer cells, with the assistance of a neighboring *cis*-acting lincRNA, TCF4/β-catenin complex was recruited to the *Ascl2* enhancer immediately downstream of the *Ascl2* locus to drive high-level *Ascl2* expression [30]. Moreover, β-catenin bound to enhancers and direct enhancer-promoter looping at mesendodermal (ME) lineage genes in human embryonic stem cells (hESC) [31]. To testify how canonical Wnt signaling regulate *Ascl2* expression, we performed chromatin immunoprecipitation and sequencing (ChIP-Seq) to assess the genome-wide occupancy of β-catenin in differentiated TS cells in the presence of CHIR or not. As expected, β-catenin occupied the promoter regions of the known Wnt target genes, such as Axin2 and Gcm1 (Fig. 6g). However, β-catenin exhibited enhanced enrichment at a site about 20 kb upstream of the *Ascl2* promoter in the presence of CHIR (Fig. 6h), which was confirmed by ChIP-qPCR analysis (Fig. 6i). These data suggest that hyperactivation of canonical Wnt signaling might repress *Ascl2* expression through β-catenin binding to the remote upstream regions of the *Ascl2* locus during trophoblast differentiation.

## Discussion

The canonical Wnt-β-catenin signaling is essential for a variety of biological processes, including embryogenesis, stem cell maintenance and differentiation, even the cell self-renewal of cancer stem cells [32–34]. Loss or hyperactivation of canonical Wnt signaling often leads to disturbed developmental processes or even diseases [32, 35]. We provide herein genetic and molecular evidence that hyperactivation of canonical Wnt signaling disturbs trophoblast differentiation in the EPC via repression *Ascl2* expression during placental development. These findings highlight the necessity of suitable Wnt signaling during trophoblast differentiation.

Wnt signaling has been reported to regulate the proliferation and differentiation of stem and progenitor cells, during the processes of both embryonic development and adult tissue homeostasis [34, 36], and aberrant Wnt signaling underlies various human diseases [32]. Human colon cancer with *APC* mutation, rendering inappropriate β-catenin stabilization and hyperactivated canonical Wnt pathway [37], exhibited a hereditary cancer syndrome named familiar adenomatous polyposis (FAP) [38, 39]. Excessive activation of Wnt signaling led to pathological bone deposition and hardening [32]. As to placental development, previous studies showed that the deletion of several members of Wnt signaling pathway impaired placental development [9–15]. Moreover, Fzd5-mediated canonical Wnt signaling has been proven essential for trophoblast syncytialization in both human and mouse [9]. Recent findings demonstrate the necessity of Wnt signaling for the derivation and maintenance of hTSCs [17] and organoids [16, 18]. However, excessive Wnt signaling disturbs development and functions of placental trophoblast cells. In complete hydatidiform mole (CHM) placenta, elevated expression of nuclear β-catenin in the extravillous trophoblasts (EVT) were observed, indicating that aberrant hyperactivation of Wnt pathway contributes to abnormal invasive trophoblast differentiation and invasion [19]. Moreover, inactivation of negative regulators of Wnt signaling, such as APC and SFRP2, was found in choriocarcinoma cells, suggesting that higher activity of Wnt pathway is involved in the progress and function of trophoblast cancers [40, 41]. On the basis of the findings, we wonder what would occur if the activity of Wnt signaling were elevated abnormally during placental development. In the present study, employing different genetic mouse models (*Sfrp1* and *Sfrp5* dKO mice, *Prm*-*cre*; *Ctnnb1*^*f(EX3)/+*^ global null mice, and *CYP19*-*cre*; *Ctnnb1*^*f(EX3)/+*^ trophoblast-specific null mice) that render hyperactivation of canonical Wnt-β-catenin pathway, we found that increased activity of canonical Wnt pathway promoted the excessive differentiation of TGCs via downregulating *Ascl2* expression. In addition, since canonical Wnt activity has been reported to promote trophoblast invasion [42], hyperactivation of canonical Wnt signaling in the differentiated TGCs might abnormally promote the invasive ability of these TGCs, leading to their excessive invasion into the maternal decidua, which may help explain the pathogenesis of human accrete pregnancy and worth further investigation.

In addition, as the inhibitors of Wnt pathway, loss of *Sfrp1,5* induced hyperactivation of canonical Wnt signaling which impaired trophoblast differentiation, suggesting that the mechanism that keeps the activity of canonical Wnt signaling in a suitable state to maintain the niche for trophoblast differentiation and TGC development, exists in vivo during normal placentation. In order to maintain the niche, a complicated signaling network is needed to preserve the balance of trophoblast differentiation.

*Ascl2* is essential for the maintenance of the EPC during early placental development in mice [5]. *Ascl2* knockout and hypomorphic mouse model showed abnormal placentation with decreased EPC layer and/or spongiotrophoblast cell lineage as well as increased number of TGCs [5, 43]. In the present study, we found the similar phenomenon when canonical Wnt signaling was hyperactivated and *Ascl2* expression was repressed. However, little is known concerning how canonical Wnt signaling regulates *Ascl2* expression. In most cases, the canonical Wnt-β-catenin signaling stimulates the transcription of target genes, such as *c-Myc* [44], *Axin2* [45] and *Gcm1* [9, 15], through promoter binding by the transcriptional activator complex containing LEF/TCF and β-catenin. In the present work, we observed that β-catenin exhibited enhanced enrichment at the site about 20 kb upstream of Ascl2 locus upon CHIR treatment. However, TCF/LEF might be not required for β-catenin-mediated Ascl2 suppression (Additional file 1: Fig. S8). Since β-catenin has been reported to bind to the downstream or upstream enhancer to promote the expression of its target genes [30, 31], we speculated that excessive canonical Wnt signaling might regulate *Ascl2* expression in a similar manner, but to repress *Ascl2* expression. Moreover, recent findings have demonstrated that canonical Wnt pathway inhibits the expression of its target genes, including the tumor suppressor 15-prostaglandin dehydrogenase [46], *RANKL* [47] and E-cadherin [48]. However, the exact mechanism via which canonical Wnt signaling represses gene expression requires further exploration.

The chorionic development was also impaired, even though the expression of Gcm1, essential for chorionic trophoblast differentiation [49] and regulated by canonical Wnt signaling [9], was increased upon the hyperactivation of canonical Wnt pathway (Fig. 5e). One possibility is that while canonical Wnt signaling is essential for chorionic trophoblast differentiation, hyperactivation of canonical Wnt signaling which increases Gcm1 expression, is not necessarily beneficial for terminal differentiation of chorionic trophoblast. This could be explained by the fact that Gcm1 overexpression just induces a rapid arrest of trophoblast proliferation and restricts their differentiation fate towards syncytiotrophoblast cells, but is not sufficient to render cell shape changes or cell-cell fusion during subsequent terminal differentiation and syncytium formation. Another possibility is that the disturbed EPC differentiation with reduced *Ascl2* expression resulting from hyperactivated canonical Wnt signaling, led to defective chorionic trophoblast differentiation, since the mouse models with *Ascl2* deletion [5] or reduced *Ascl2* expression [50] also displayed disturbed chorionic/labyrinth development. Even though it has been shown that *Ascl2*-expressing trophoblast cells was not required for labyrinth development [51], this work is not imprecisely, since the wildtype trophoblast could provide the labyrinth layer with *Ascl2*-expressing trophoblast cells. In addition, the EPC/spongiotrophoblast layer might functions as structural support for the chorionic/labyrinth development, since *Ascl2*-expressing trophoblast cells are also present in the chorion and subsequent labyrinth layer.

## Conclusions

In summary, hyper-activation of the canonical Wnt signaling, achieved by either the deletion of *Sfrp1,5* or the expression of a stabilized β-catenin globally and trophoblast-specifically, resulted in impaired EPC trophoblast differentiation and excessive TGC expansion. Further exploration using the trophoblast stem cell line uncovered that hyper-activated Wnt pathway led to the repression of *Ascl2*, which is important for trophoblast differentiation. This study provides genetic and molecular evidence that appropriate canonical Wnt pathway is crucial for EPC trophoblast differentiation, which is of high clinical significance since there is a strong correlation between abnormal Wnt signaling and human trophoblast diseases [8].

## Materials and methods

### Animals and tissue collection

*Ctnnb1*^*f(EX3)/f(EX3)*^ mice, *Sfrp1* and *Sfrp5* double mutant mice, and *Cyp19-cre* transgenic mice were generated as previously described [25, 52–54]. *Prm-cre* transgenic mice were obtained from Jackson Laboratory. *Ctnnb1*^*f(EX3)/f(EX3)*^ mice were mated with *Prm-cre* mice to get mice with global hyperactivation of the canonical Wnt pathway. *Ctnnb1*^*f(EX3)/f(EX3)*^ mice were mated with *Cyp19-cre* transgenic mice to get trophoblast-specific hyperactivation of canonical Wnt signaling. Eight-week females were mated with fertile males to induce pregnancy and the day when virginal plugs were seen was considered as embryonic day 0.5 (E0.5). Pregnant females were sacrificed and implantation sites were weighted, frozen, or fixed in 10% neutral buffered formalin. Tail genotyping of the embryos and newborns were determined by PCR.

### Histological analysis and immunostaining

Histological and immunostaining analysis were performed as described previously [9]. In brief, dissected implantation sites were fixed in 10% neutral buffered formalin at room temperature overnight. Tissues underwent dehydration using graded ethanol, vitrification by dimethylbenzene and were embedded in paraffin, and 5 μm transverse sections were used for hematoxylin and eosin (H&E) staining, immunohistochemistry (IHC), and immunofluorescence (IF). For frozen tissue, 10 μm transverse sections were used for immunofluorescence (IF). Antibodies used for immunohistochemistry (IHC) and immunofluorescence (IF) include: cytokeratin (DAKO, Z0622, 1:200), active β-catenin (CST, #8814, 1:1000), β-catenin (Abcam, ab6302, 1:1000), Plf (Santa Cruz, sc47347, 1:200), and Pcdh12 (MAB7926, 1:200).

### In situ hybridization

In situ hybridization with isotopes-labeled antisense RNA probes was performed on cryosections (10 μm) as previously described [55]. Whole-mount in situ hybridization with digoxygenin (DIG)-labeled antisense RNA probes was conducted using standard procedures. The primers for probe production are listed in Additional file 1: Table S1.

### Trophoblast stem cell derivation, culture, and differentiation

*Sfrp1* and *Sfrp 5* dKO as well as WT TS cells were derived from E3.5 mouse blastocysts as described previously [28]. Briefly, blastocysts were obtained from E3.5 uterus, and transferred to four-well tissue culture dish containing the mitomycin C-treated MEF feeders in TS medium+F4H [RPMI 1640 (Gibco, 31870082) containing 20% FBS (Gibco, 16000-044), 1 mM sodium pyruvate, 100 μM β-mercaptoethanol, 2 mM L-glutamine, 25 ng/ml FGF4 (Peprotech, 100-31), and 1 mg/ml Heparin (Sigma, 2,608,411)] and cultured at 37 °C, 5% CO_2_. Then, the blastocysts hatched from zona pellucida and attached to the wells to form outgrowth. After disaggregation of the blastocyst outgrowth, feed cells with 70%FCM + 1.5xF4H (30% TS medium, 70% mouse fibroblast-conditioned medium, 1.5x FGF4/Heparin) for early passages and with 70%FCM + 1xF4H for maintenance. For TS cells differentiation, TS cell medium was used without the supplementation of FGF4, heparin, and FCM. For the treatment of TSC, CHIR99021 (Biovision, 1748-5), XAV-939 (MedChemExpress, HY-15147), and iCRT3 (MedChemExpress, HY-103705) were used.

### Quantitative real-time PCR

RNA extraction and quantitative RT-PCR was performed as described [9]. Briefly, cells were directly lysed in RNAiso plus (TAKARA, 9109) after pumping out the medium. RNA was extracted in the upper layer after chloroform added and centrifuged. Purified RNA could be obtained after precipitation with isopropanol and washing with ethanol. cDNA was reverse transcripted with PrimeScript™ RT reagent Kit with gDNA Eraser (Perfect Real Time) (TAKARA, RR047A) according to the manufacturer’s instructions. qRT-PCR were performed with TB Green® Premix Ex Taq™ II (Tli RNaseH Plus) (TAKARA, RR820A). All assays were performed at least three times. The primers for real-time PCR are all listed in Additional file 1: Table S1.

### Western blotting

Protein extraction and Western blotting were performed as described previously [9]. Antibodies used for western blotting include active β-catenin (CST, #8814, 1:1000), β-catenin (Abcam, ab6302, 1:1000), and β-actin (Bioworld, AP0063, 1:5000).

### RNA-seq and data analysis

RNA from *Cyp19-cre* and *Cyp19-cre*; *Ctnnb1*^*f(EX3)/+*^ placentas at E8.5 were extracted using RNeasy Micro Kit (QIAGEN, 74004) according to the manufacturer’s instructions. Purified RNA was prepared using TruSeq® RNA Sample Preparation V2 (Illumina, RS-122-2001) and subjected to 50-bp single-end sequencing with a BGISEQ-500 sequencer. RNA-seq raw data were initially filtered to obtain clean data after quality control. Clean data were aligned to the mouse genome (mm10) by HISAT2. Raw counts for each gene were calculated by EdgeR. DEGs were defined as genes with *P* value less than 0.05 and fold change larger than 1.5.

### ChIP-Seq and ChIP-qPCR

ChIP was performed according to the reported previously [56]. Briefly, about 2 × 10^6^ TS cells were cross-linked with 16% formaldehyde (Cell Signaling Technology, 12,606) at final concentration of 1% at room temperature for 10 min and quenched with 1/10 volume of 1.25 M glycine for 15 min on ice. Cell lysate in lysis buffer III were sonicated using Bioruptor pico (Diagenode) and then incubated with 4 μg non-phospho (active) β-catenin antibody (Cell Signaling Technology, 8814) overnight at 4°Cwith rotation. Immunoprecipitated complexes were collected with 15 μl Protein A Dynabeads (Invitrogen, 10006D) for 1 h at 4 °C with rotation. Subsequently, beads were washed sequentially once with low-salt buffer, twice with high-salt buffer, once with LiCl buffer, twice with TE, and then eluted in 400 μl of elution buffer for 30 min at 65 °C with shaking. The eluates were incubation at 65 °C for 8 h to reverse the cross-linking. Next, eluates were treated with proteinase K for 1 h at 55 °C and then RNase A for 30 min at 37 °C before DNA was extracted and purified. The ChIP libraries were prepared according to the instruction manual using KAPA DNA HyperPrep Kits (Roche, KK8502) and then run on the Illumina sequencer Hiseq-Xten PE150. Primers for qPCR were listed in Additional file 1: Table S1.

### ChIP-Seq analysis

The primary analysis of ChIP-Seq datasets were performed by using Illumina’s Genome Analysis pipeline. The sequencing reads were aligned to the mouse genome (mm10) by HISAT2. Only uniquely aligned reads were kept. MACS2 was applied for peak call using default parameters.

### Statistical analysis

Statistical analysis was performed with the GraphPad Prism 8 software. In data that was normally distributed, Student’s *t* test was performed to determine the significance of a difference between two groups. When comparing the means of more than two groups, a one-way ANOVA was used. Data were presented as means ± SEM in all experiments unless otherwise indicated. *P < 0.05 was considered to indicate a significant result.

## Supplementary information

**Additional file 1: : Figure S1.** The expression of *Sfrp1*, *Sfrp2* and *Sfrp5* during early placental development. A The expression of *Sfrp1*, *Sfrp2*, and *Sfrp5* was analyzed by whole-mount in situ hybridization at E7.5 and E8.5. B The expression of *Sfrp1* and *Sfrp5* was detected by in situ hybridization at E7.5-E9.5. The signals were pink. Images in (A) and (B) are representatives of at least two independent experiments. **Figure S2.** Statistical description of the phenotype of the *Sfrp1* and *Sfrp5* dKO placenta. A Statistical analysis of spongiotrophoblast thickness and TGCs number in WT and dKO placenta on E9.5. B Quantification of active-β-catenin signal intensity in Fig. 2a. *, *P* < 0.05. **Figure S3.** Nuclear localization of active-β-catenin increased in the placenta of *Ctnnb1*^*Δ/+*^ conceptus. A Immunohistochemistry of active-β-catenin in *Ctnnb1*^*f(EX3)/+*^ and *Ctnnb1*^*Δ/+*^ placenta on E9.5. B Quantification of active-β-catenin signal intensity in (A). *, P < 0.05. **Figure S4.** Global stabilization of β-catenin leads to impaired trophoblast development and embryonic lethality. HE and CK staining of the sections of *Ctnnb1*^*f(EX3)/+*^ and *Ctnnb1*^*Δ/+*^ conceptus on E7.5. Images are representatives of at least three independent experiments. Dec, decidua; EPC, ectoplacental cone; Em, embryo; TGC, trophoblast giant cell. **Figure S5.** Trophoblast-specific stabilization of β-catenin induces hyperactivation of canonical Wnt pathway. A The localization of active-β-catenin was revealed by immunostaining at E7.5. Cy3-labeled active β-catenin in red, DAPI-labeled nuclei in blue. Images are representatives of at least three independent experiments. B Quantification of active-β-catenin signal intensity in (A). *, P < 0.05. **Figure S6.** Decreased genes with trophoblast-specific stabilization of β-catenin are related to cell cycles. KEGG analysis of the decreased genes (A, B) between *Cyp 19-cre* and *Cyp19-cre*; *Ctnnb1*^*f(Ex3)/+*^ placentas (Fold change>1.5, *P* value < 0.05). **Figure S7.** *Sfrp1* and *Sfrp5* deficiency led to increased activity of Wnt signaling. A TOP-Flash assay in the WT and dKO TS cells. B The expression of *Ascl2*, *Tpbpa* and *Pl1* in dKO TS cells differentiated for 4 days, in the presence of XAV-939 or not. *, P < 0.05. C QRT-PCR analysis of the expression of *Ascl2*, *Tpbpa* and *Pl1* in dKO TS cells differentiated for 4 days, with *Ascl2* overexpression or not. **Figure S8.** TCF/LEF might be not required for β-catenin-mediated *Ascl2* suppression. A The expression of *Ascl2* in WT TS cells treated with indicated conditions. iCRT3, a small molecule that abrogates β-catenin–TCF interaction. *, P < 0.05. ns, not significant. B The top 13 conserved transcription factors binding motif at the β-catenin binding site, 20 kb upstream of the Ascl2 gene. **Table S1.** Primers Information.

**Additional file 2: : Table S2.** Differentially expressed genes (DEG) between the *Cyp19-cre* and *Cyp19-cre; Ctnnb1*^*f(Ex3)/+*^ placentas (fold change > 1.5). **Table S3.** Genes expressed in the *Cyp19-cre* and *Cyp19-cre; Ctnnb1*^*f(Ex3)/+*^ placentas.

## Data Availability

All sequencing data including RNA-seq and ChIP-seq data have been deposited in the Gene Expression Omnibus (GEO) database with the accession codes GSE146433 (https://www.ncbi.nlm.nih.gov/geo/query/acc.cgi?acc=GSE146433) and GSE146432 (https://www.ncbi.nlm.nih.gov/geo/query/acc.cgi?acc=GSE146432) [57], respectively. If any additional information and materials are needed, they will be available upon request from the corresponding authors.

## Abbreviations

*Ascl2*: achaete-scute complex homolog-like 2 (also known as *Mash2*)
EPC: Ectoplacental cone
ExE: Extraembryonic ectoderm
*Gcm1*: Glial cells missing-1
*Hand1*: Heart and neural crest derivatives expressed transcript 1
TGC: Trophoblast giant cell
