# Supplementary material for: Hyperactivated Wnt-β-catenin signaling in the absence of sFRP1 and sFRP5 disrupts trophoblast differentiation through repression of Ascl2
Source: BMC Biol. 2020 Oct 27;18:151. doi: 10.1186/s12915-020-00883-4 (PMC7592576; doi:10.1186/s12915-020-00883-4)
Supplement: Supplementary file 1 — Additional file 1: : Figure S1. The expression of Sfrp1, Sfrp2 and Sfrp5 during early placental development. A The expression of Sfrp1, Sfrp2, and Sfrp5 was analyzed by whole-mount in situ hybridization at E7.5 and E8.5. B The expression of Sfrp1 and Sfrp5 was detected by in situ hybridization at E7.5-E9.5. The signals were pink. Images in (A) and (B) are representatives of at least two independent experiments. Figure S2. Statistical description of the phenotype of the Sfrp1 and Sfrp5 dKO placenta. A Statistical analysis of spongiotrophoblast thickness and TGCs number in WT and dKO placenta on E9.5. B Quantification of active-β-catenin signal intensity in Fig. 2a. *, P < 0.05. Figure S3. Nuclear localization of active-β-catenin increased in the placenta of Ctnnb1Δ/+ conceptus. A Immunohistochemistry of active-β-catenin in Ctnnb1f(EX3)/+ and Ctnnb1Δ/+ placenta on E9.5. B Quantification of active-β-catenin signal intensity in (A). *, P < 0.05. Figure S4. Global stabilization of β-catenin leads to impaired trophoblast development and embryonic lethality. HE and CK staining of the sections of Ctnnb1f(EX3)/+ and Ctnnb1Δ/+ conceptus on E7.5. Images are representatives of at least three independent experiments. Dec, decidua; EPC, ectoplacental cone; Em, embryo; TGC, trophoblast giant cell. Figure S5. Trophoblast-specific stabilization of β-catenin induces hyperactivation of canonical Wnt pathway. A The localization of active-β-catenin was revealed by immunostaining at E7.5. Cy3-labeled active β-catenin in red, DAPI-labeled nuclei in blue. Images are representatives of at least three independent experiments. B Quantification of active-β-catenin signal intensity in (A). *, P < 0.05. Figure S6. Decreased genes with trophoblast-specific stabilization of β-catenin are related to cell cycles. KEGG analysis of the decreased genes (A, B) between Cyp 19-cre and Cyp19-cre; Ctnnb1f(Ex3)/+ placentas (Fold change>1.5, P value < 0.05). Figure S7. Sfrp1 and Sfrp5 deficiency led to increa [file 12915_2020_883_MOESM1_ESM.docx]

**Supplementary Information**

**Supplemental Figures and Legends**


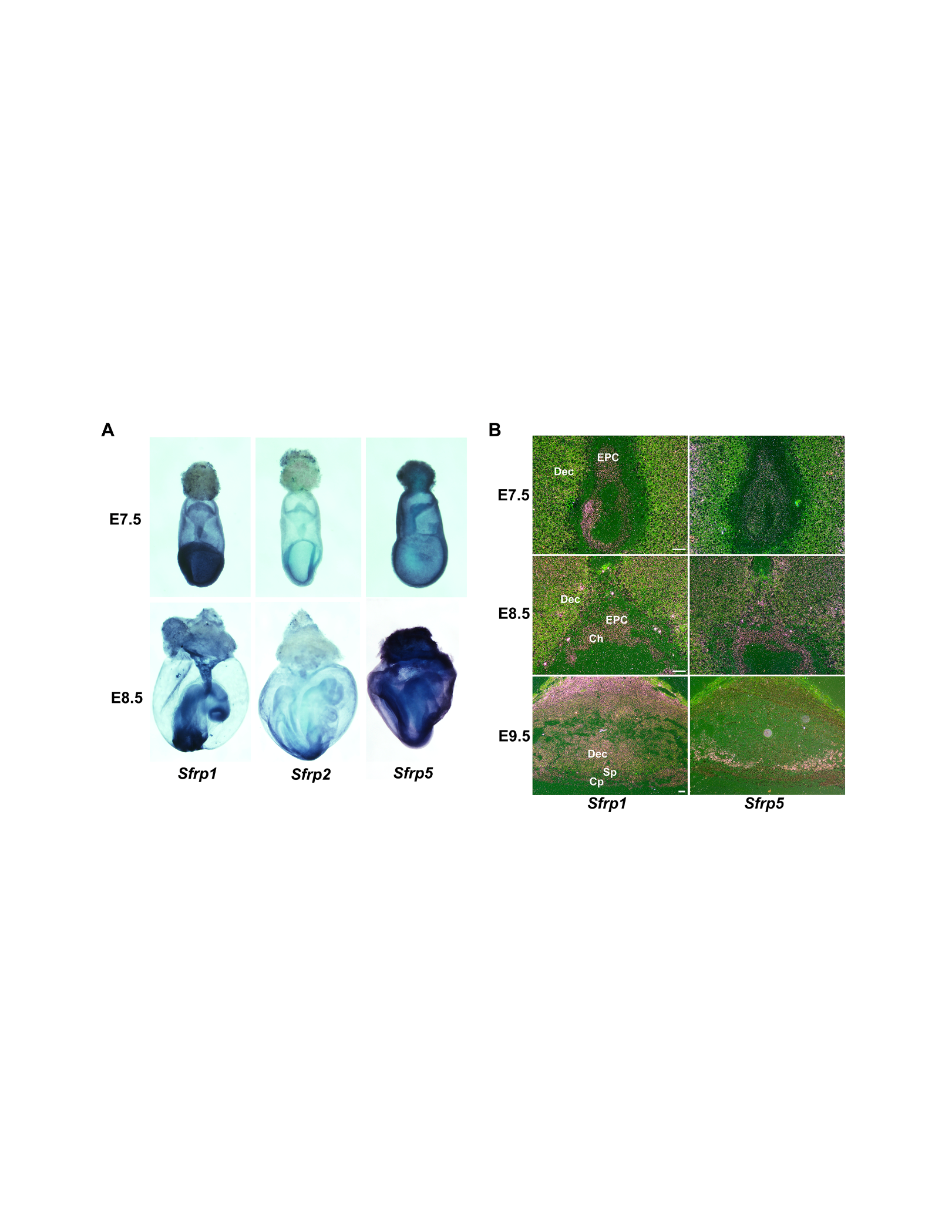


**Figure S1. The expression of *Sfrp1*, *Sfrp2* and *Sfrp5* during early placental development.** **A** The expression of *Sfrp1*, *Sfrp2*, and *Sfrp5* was analysed by whole-mount *in situ* hybridization at E7.5 and E8.5. The signals were blue. **B** The expression of *Sfrp1* and *Sfrp5* was detected by *in situ* hybridization at E7.5-E9.5. The signals were pink. Images in (**A**) and (**B**) are representatives of at least two independent experiments. EPC, ectoplacental cone; Dec, decidua; Ch, chorion; Cp, chorionic plate; Sp, spongiotrophoblast. Scale bar, 100 μm.

**
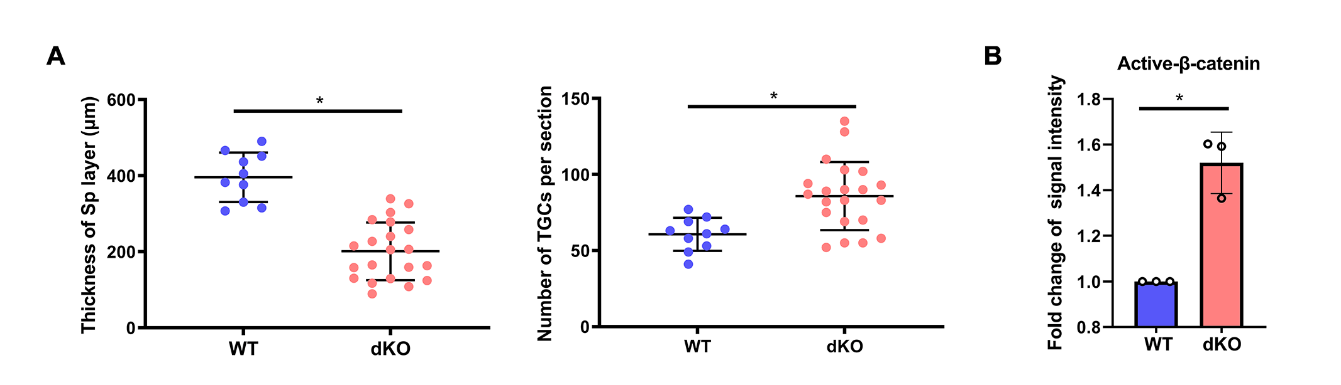
**

**Figure S2. Statistical description of the phenotype of the *Sfrp1* and *Sfrp5* dKO placenta. A** Statistical analysis of spongiotrophoblast thickness and TGCs number in WT and dKO placenta on E9.5. **B** Quantification of active-β-catenin signal intensity in Figure 2A. *, P<0.05.

**
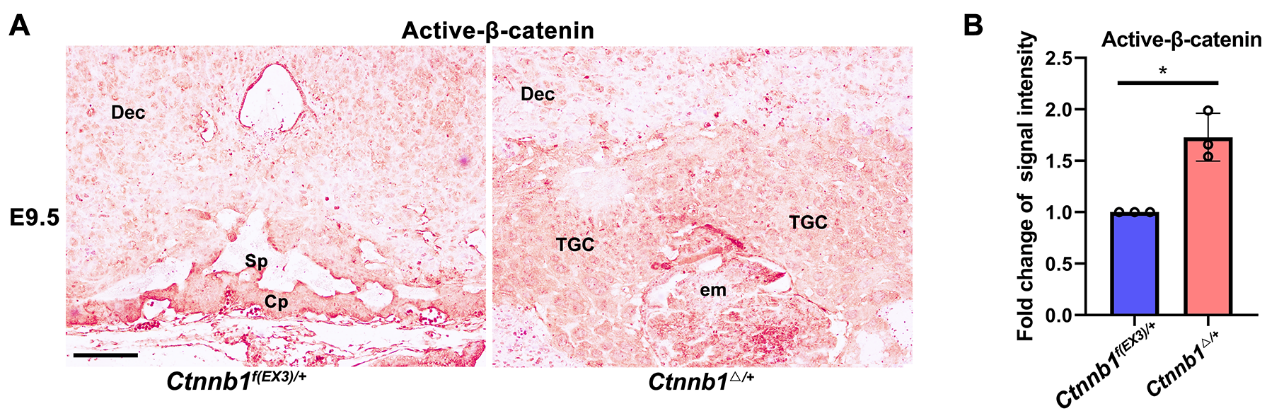
Figure S3. Nuclear localization of active-β-catenin increased in the placenta of *Ctnnb1^Δ/+^* conceptus. A** Immunohistochemistry of active-β-catenin in *Ctnnb1^f(EX3)/+^* and *Ctnnb1^Δ/+^* placenta on E9.5. Dec, decidua; Sp, spongiotrophoblast; Cp, chorionic plate; TGC, trophoblast giant cell; em, embryo. **B** Quantification of active-β-catenin signal intensity in (**A**). *, P<0.05.

**
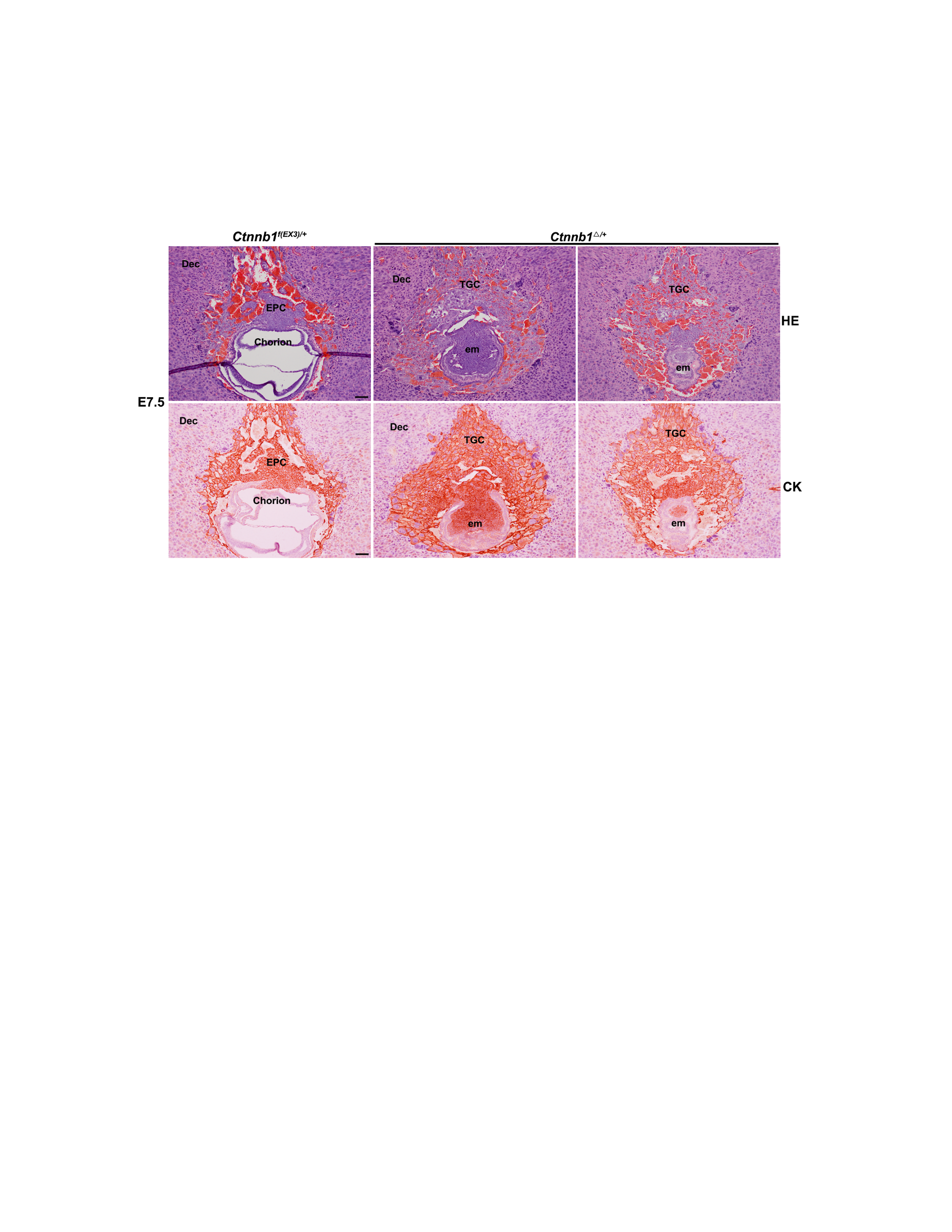
**

**Figure S4.** **Global stabilization of β-catenin leads to impaired trophoblast development and embryonic lethality.** HE and CK staining of the sections of *Ctnnb1^f(EX3)/+^* and *Ctnnb1^Δ/+^* conceptus on E7.5. Images are representatives of at least three independent experiments. Dec, decidua; EPC, ectoplacental cone; Em, embryo; TGC, trophoblast giant cell. Scale bar, 100 μm.


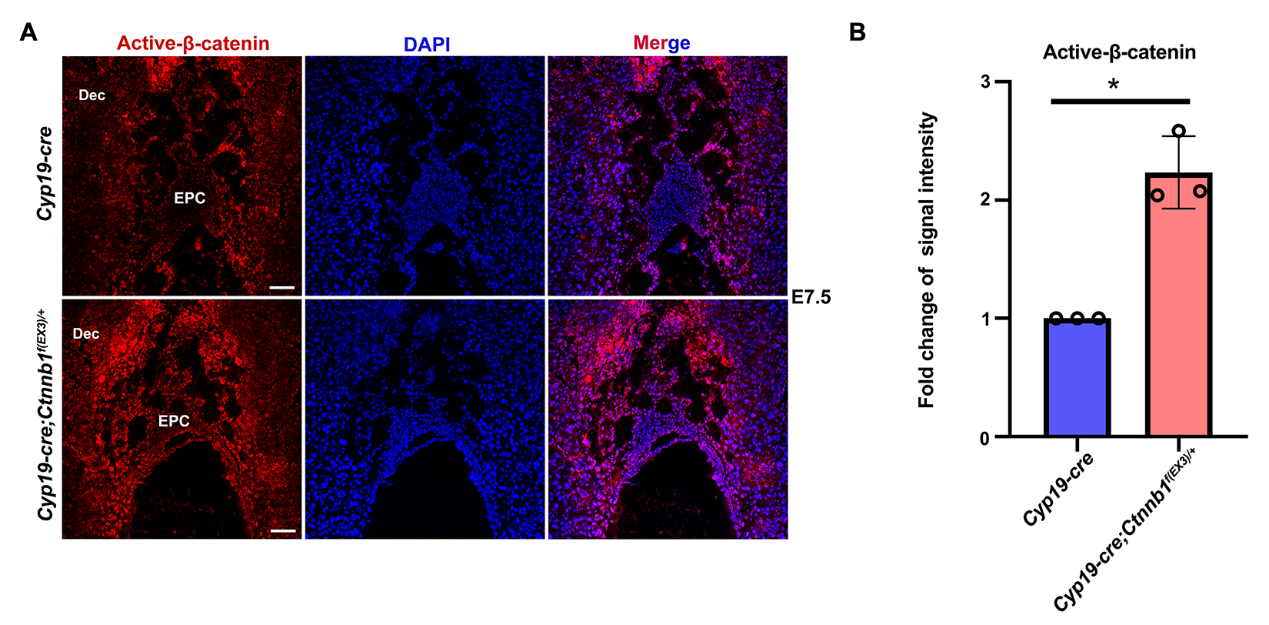


**Figure S5.** **Trophoblast-specific stabilization of β-catenin induces hyperactivation of canonical Wnt pathway. A** The localization of active-β-catenin was revealed by immunostaining at E7.5. Cy3-labeled active β-catenin in red, DAPI-labeled nuclei in blue. Images are representatives of at least three independent experiments. Dec, decidua; EPC, ectoplacental cone. Scale bar, 100 μm. **B** Quantification of active-β-catenin signal intensity in (**A**). *, P<0.05.


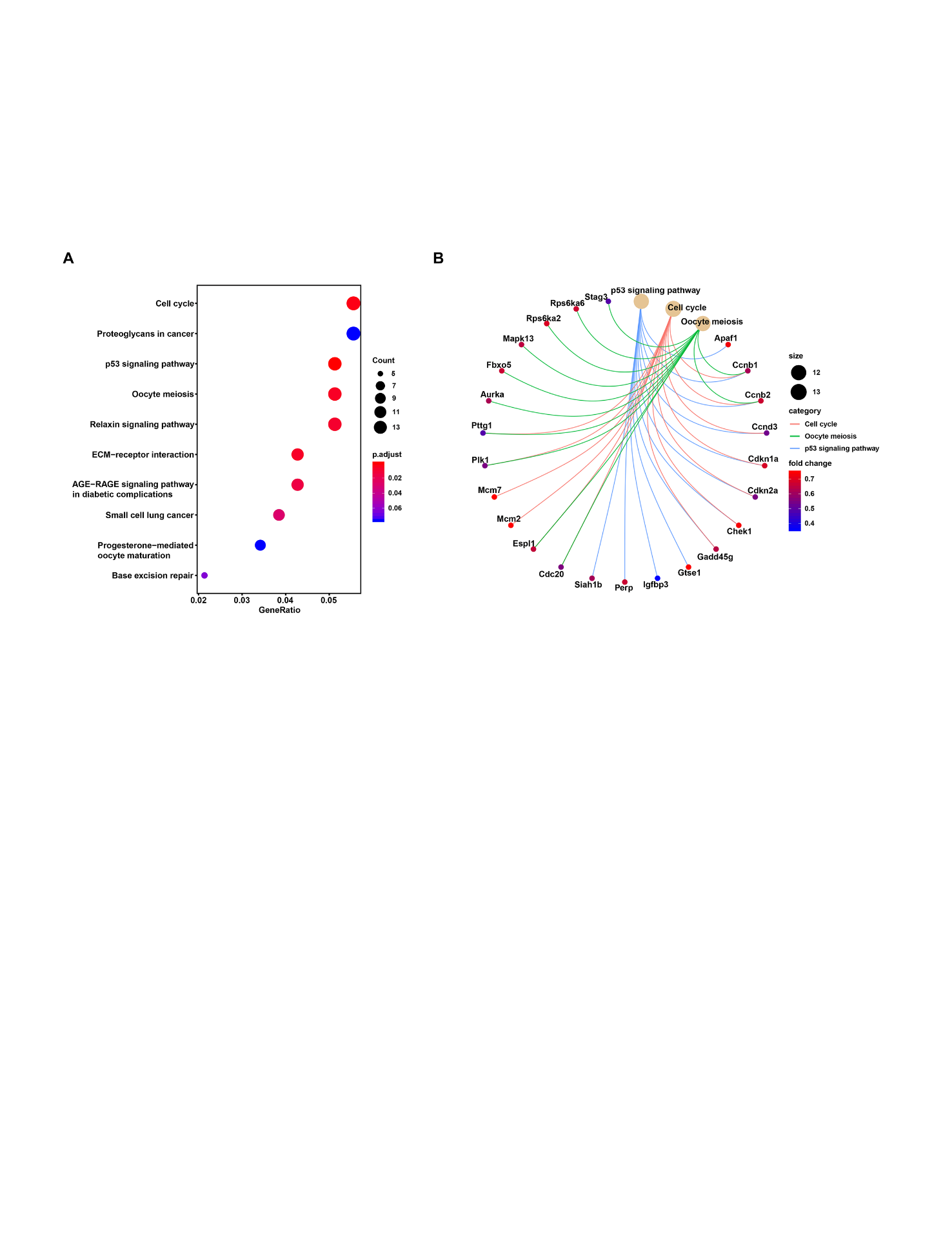


**Figure S6. Decreased genes with trophoblast-specific stabilization of β-catenin are related to cell cycles.** KEGG analysis of the decreased genes (**A**, **B**) between *Cyp19-cre* and *Cyp19-cre*; *Ctnnb1^f(Ex3)/+^* placentas (Fold change＞1.5, P value < 0.05).

**
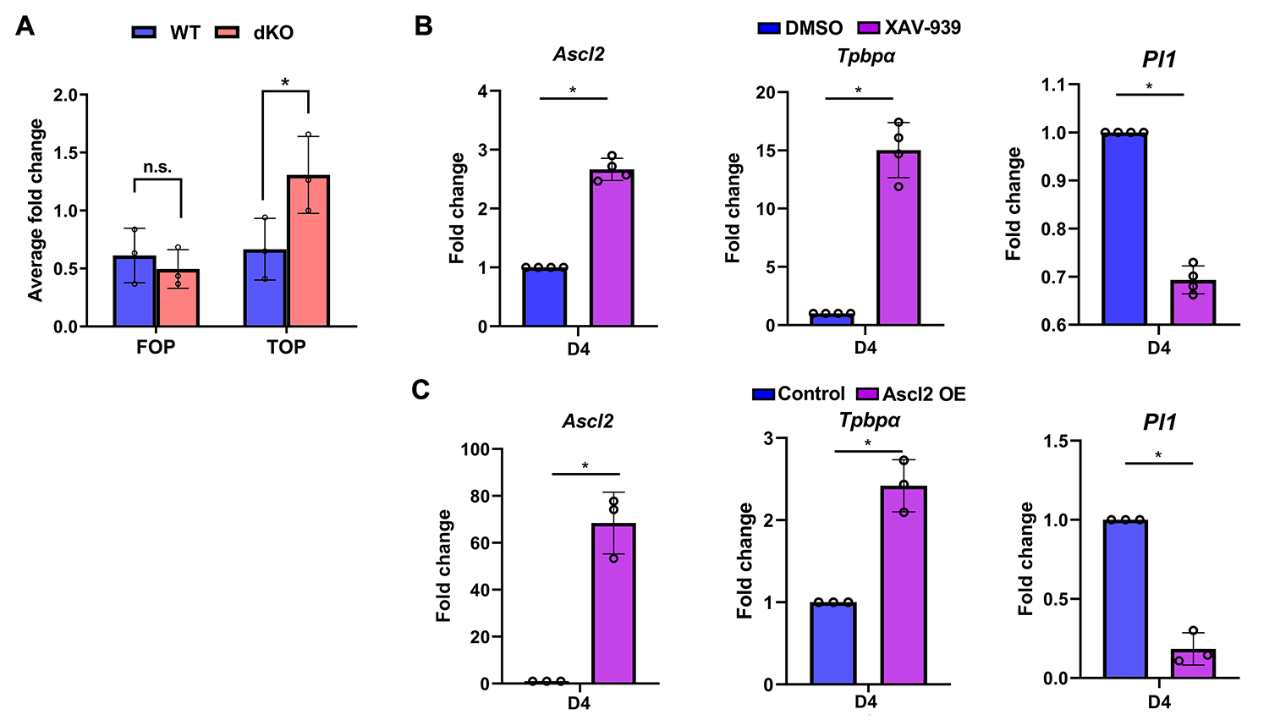
**

**Figure S7. *Sfrp1* and *Sfrp5* deficiency led to increased activity of Wnt signalling. A** TOP-Flash assay in the WT and dKO TS cells. **B** The expression of *Ascl2*, *Tpbpa* and *Pl1* in dKO TS cells differentiated for 4 days, in the presence of XAV-939 or not. *, P<0.05. **C** QRT-PCR analysis of the expression of *Ascl2*, *Tpbpa* and *Pl1* in dKO TS cells differentiated for 4 days, with *Ascl2* overexpression or not.


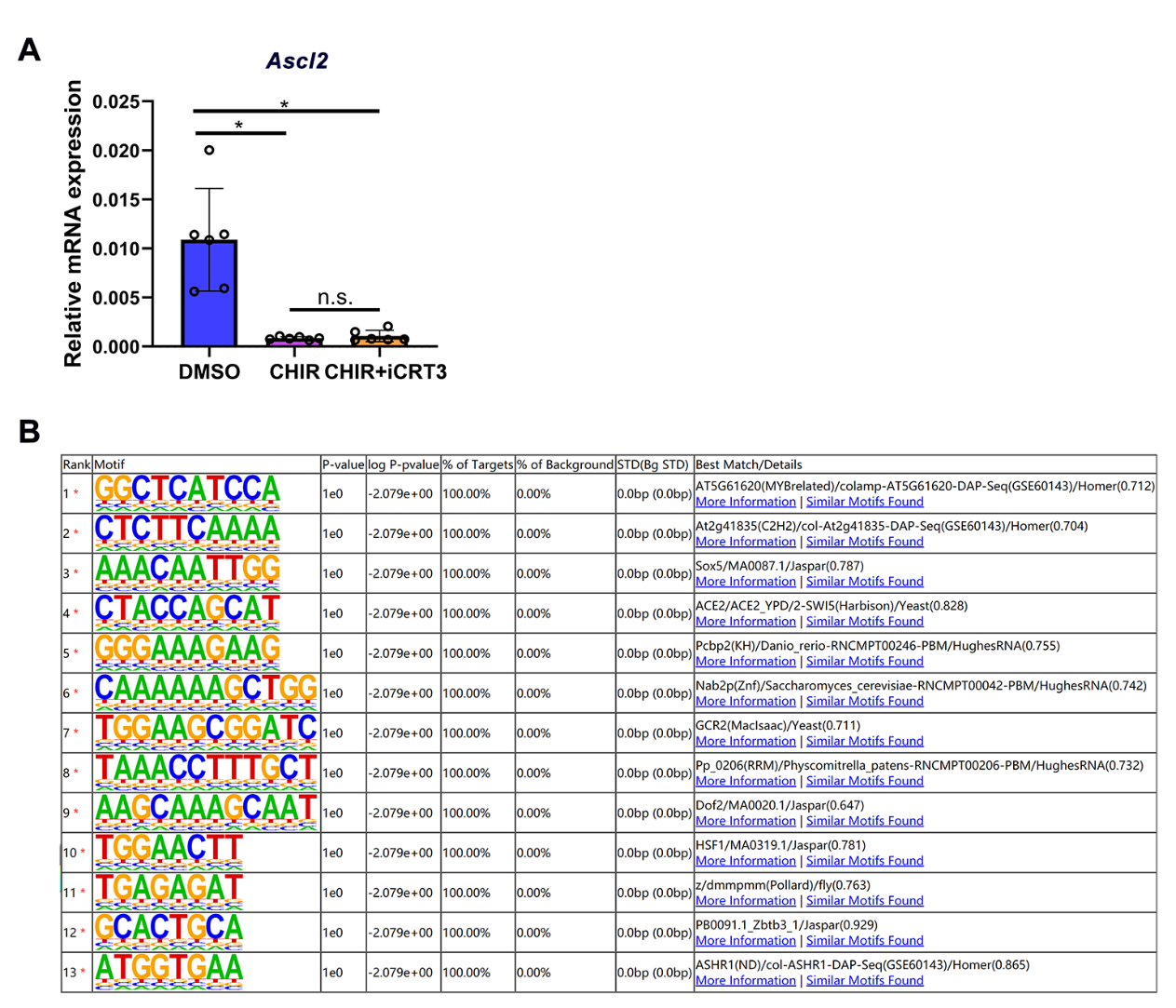


**Figure S8. TCF/LEF might be not required for β-catenin-mediated *Ascl2* suppression. A** The expression of *Ascl2* in WT TS cells treated with indicated conditions. iCRT3, a small molecule that abrogates β‐catenin–TCF interaction. *, P<0.05. ns, not significant. **B** The top 13 conserved transcription factors binding motif at the β-catenin binding site, 20 kb upstream of the Ascl2 gene.

**Table S1 Primers Information.**

| Name | Sequence (5’→3’) |
| --- | --- |
| **Quantitative RT-PCR primers** | |
| Ascl2 (Mash2)-F | CAGCTGCGAGGGAGAGCTAA |
| Ascl2 (Mash2)-R | GATGCTCAGTAGCCCCCTAACC |
| Hand1-F | GGGTTAAACCCGGTCTTTGG |
| Hand1-R | AAGGACCTGCCGACCTCTTG |
| Tpbpα-F | CAGAGAGTGGCGATGGGTTTT |
| Tpbpα-R | GACAATGGCACAGTGGCTGTT |
| Pl1-F | CAGGCTCCGGAATGCAATT |
| Pl1-R | GCAGTTGGTTTGGAGGACACA |
| Vegfa-F | GTGACTCCATGGCCCTCACTT |
| Vegfa-R | CGTCCTCACCTTCGCGTTTA |
| Mmp9-F | CACCGAGCTATCCACTCATCAA |
| Mmp9-R | TGGTTTCAGCAGATTTACAGGAC |
| Ppard-F | TTTGCTGTCAAGTTCAATGCG |
| Ppard-R | GTACTGGCTGTCAGGGTGGTT |
| Gcm1-F | GGTGCTTGAGTGGGCCGATCC |
| Gcm1-R | AGGTGCCGCTGCGCATTCTT |
| **Primers for *in situ* hybridization probes production** | |
| sFrp1-F | GTTGCGGACCTGTGAGGATTT |
| sFrp1-R | GCCTGGAACTGGGCTTAGACT |
| sFrp2-F | GTTCCTGTGCTCGCTCTTCG |
| sFrp2-R | GGATTTCTTCAGGTCCCTTTCG |
| sFrp5-F | GGGGACCGAAAGTTGATTGG |
| sFrp5-R | CCTGTGCAGTAGGTGCGTGAA |
| Pl1-F | TTCCTCACTTGGAGCCTACA |
| Pl1-R | CTCTACATAACTGAGGAGGG |
| Tpbpα-F | TCCAAGGACCTCTGAAGAGC |
| Tpbpα-R | AGGATCCCACTTGTCAGGGG |
| **Primers for the ChIP-qPCR** | |
| Ascl2 (Mash2)-20kb-F | TGGGATTTAGAATCACCACAGAA |
| Ascl2 (Mash2)-20kb-R | CTAAGAAGCAGAGCAAGATGGGT |
